# Burnout among pharmacists in Tunisia during COVID-19 pandemic

**DOI:** 10.1192/j.eurpsy.2022.1265

**Published:** 2022-09-01

**Authors:** D. Falfel, D. Cherif, H. Falfel, N. Tlili, C. Drira, M. Razgallah Khrouf, G. Hamdi, H. Ben Ammar

**Affiliations:** 1 Razi Hospital, Psychiatry Ward, Manouba, Tunisia; 2 Faculty of pharmacy, Pharmaceutical Department, Monastir, Tunisia; 3 Faculty of pharmacy, Lncm, Tunis, Tunisia; 4 Faculty of pharmacy, Pharmacology, Monastir, Tunisia

**Keywords:** pharmacist, Impact, Covid-19, burnout

## Abstract

**Introduction:**

Both public and private sector pharmacists were instrumental in containing this health crisis in Tunisia. The high workload had a considerable impact on their mental health during the outbreak of the Corona Virus.

**Objectives:**

This study aims to assess burnout and the psychological toll of the pandemic among pharmacists in Tunisia during covid-19.

**Methods:**

258 Tunisian pharmacists working in the public and private sector participated in a questionnaire. Burnout was assessed by the Maslach burnout scale. Regression analysis was used to assess the impact of the pandemic on Tunisian pharmacists.

**Results:**

80% of the respondents were women. Participants ranged in age from 22 to 62, 60% were married, 57% had at least one child, and 42% had been working for less than five years. The burnout scale revealed 76% burnout among them. Univariate linear regression showed that female gender (p = 0.014 <0.05) was associated with the development of burnout.

**Conclusions:**

The considerable prevalence of burnout among pharmacists during the COVID-19 pandemic in Tunisia can be attributed to the enormous and overwhelming responsibilities that any health care worker endured.

**Disclosure:**

No significant relationships.

